# Reduction of Carbon Dioxide to Formate at Low Overpotential Using a Superbase Ionic Liquid

**DOI:** 10.1002/anie.201507629

**Published:** 2015-09-22

**Authors:** Nathan Hollingsworth, S F Rebecca Taylor, Miguel T Galante, Johan Jacquemin, Claudia Longo, Katherine B Holt, Nora H de Leeuw, Christopher Hardacre

**Affiliations:** Department of Chemistry, University College London 20 Gordon Street, London, WC1H 0AJ (UK); School of Chemistry and Chemical Engineering, Queen's University Belfast David Keir Building, Belfast, BT9 5AG (UK) E-mail: c.hardacre@qub.ac.uk; Institute of Chemistry, University of Campinas—UNICAMP Campinas, SP (Brazil)

**Keywords:** CO_2_ reduction, electrolysis, formate, ionic liquids, superbases

## Abstract

A new low-energy pathway is reported for the electrochemical reduction of CO_2_ to formate and syngas at low overpotentials, utilizing a reactive ionic liquid as the solvent. The superbasic tetraalkyl phosphonium ionic liquid [P_66614_][124Triz] is able to chemisorb CO_2_ through equimolar binding of CO_2_ with the 1,2,4-triazole anion. This chemisorbed CO_2_ can be reduced at silver electrodes at overpotentials as low as 0.17 V, forming formate. In contrast, physically absorbed CO_2_ within the same ionic liquid or in ionic liquids where chemisorption is impossible (such as [P_66614_][NTf_2_]) undergoes reduction at significantly increased overpotentials, producing only CO as the product.

Although CO_2_ is a greenhouse gas thought to be involved in climate change,[1] it can also be considered as an abundant carbon building block for carbon neutral fuels and chemicals.[2] Electrochemical reduction is one route to achieve this goal. Indeed, the reduction of CO_2_ at low applied overpotentials with high efficiencies is a significant current challenge owing to its thermodynamic stability and kinetic inertness.[3] The high overpotential for CO_2_ reduction is related to the large reorganization energy associated with reduction of linear CO_2_ to the bent [^.^CO_2_]^−^ radical anion. Thus, a very negative reduction potential is required for the first electron reduction, that is, −1.9 V vs. NHE,[4] rendering reduction highly energy inefficient. Materials that form complexes with CO_2_ in a non-linear geometry can decrease this reorganization energy and catalyze the electrochemical CO_2_ reduction.[5]

Recently, promising results have been reported utilizing room-temperature ionic liquids (RTILs) for the electrochemical reduction of CO_2_. High CO_2_ solubility, intrinsic ionic conductivity, and wide potential windows of RTILs make them attractive solvents for CO_2_ electroreduction.[6] Initial reports on CO_2_ reduction in RTILs formed dialkyl carbonates through generation of ^.^CO_2_ radicals, which were reacted with alcohols using 1-alkyl-3-methylimidazolium ([C_*n*_mim]^+^) based RTILs with a range of non-coordinating anions.[7]

Further RTIL studies focused on CO_2_ reduction to products other than dialkyl carbonates. Rosen et al. reported the use of Ag electrodes in [C_2_mim][BF_4_], which was found to decrease the energy of formation of the [^.^CO_2_]^−^ radical anion through the complexation of CO_2_ with the [C_2_mim]^+^ cation.[8] This significantly reduced the overpotential for CO_2_ reduction to CO to <0.2 V. Furthermore, the cation was shown to suppress the competing H_2_ production reaction by forming a monolayer on the electrode.[9] Further decreases of the applied potential have been achieved by substitution of the Ag working electrode with MoS_2_, giving overpotentials as low as 0.054 V.[10] Brennecke and co-workers also showed that the anion influenced the product selectivity, with oxalate formation favored over CO in [C_2_mim][NTf_2_].[11] This change in product selectivity was also shown by Watkins and Bocarsly, where formate was produced in [C_2_mim][TFA] using Pb and Sn working electrodes.[12] Therein, no evidence was found for a [C_2_mim]^+^-CO_2_ complex;[13] however, the RTIL was thought to stabilize intermediates in formate production.

Although interesting, CO_2_ reduction in non-coordinating [C_*n*_mim]^+^ based RTILs is of limited applicability, owing to low CO_2_ solubility. For example, CO_2_ solubility in [C_4_mim][NTf_2_] is <0.04 CO_2_ mole fraction at 10 °C and 0.1 MPa.[14] The only coordinating IL used to date for CO_2_ reduction studies is [C_4_mim][OAc],[15] which has a CO_2_ solubility of 0.274 CO_2_ mole fraction at 25 °C and 0.1 MPa.[16] The low solubility affects the rate of product formation and limits the industrial significance of these systems.

These low solubilities have been overcome by the use of superbasic ionic liquids. In these systems, [P_66614_][124Triz], for example, has been shown to absorb equimolar quantities of CO_2_ through the chemical interaction of CO_2_ with the anion and physical absorption of CO_2_ in the solution free space (Scheme 1).[6b, 17] This set of ILs have been studied extensively for CO_2_ capture but, to date, no reports of their use in CO_2_ conversion have been published. A key feature of the anion–CO_2_ interaction is that the CO_2_ chemically binds without prior reduction to [^.^CO_2_]^−^. Notably, since CO_2_ is transformed from a linear to bent geometry on binding to the anion, this can significantly lower the CO_2_ reduction potential.

**Scheme 1:**
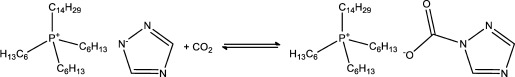
Addition of CO_2_ to [P_66614_][124Triz], showing binding of CO_2_ to the triazolide anion.

In this study, we report the first electrochemical reduction of CO_2_ captured within the superbasic RTIL [P_66614_][124Triz], providing a new low-energy pathway for CO_2_ reduction in RTILs. Full product characterization of solution and gas phase products is reported using ^1^H NMR spectroscopy and online GC analysis, respectively.

The influence of the anion identity on CO_2_ reduction was examined by recording cyclic voltammograms (CVs) at a Ag working electrode in 0.1 mol L^−1^ [P_66614_][NTf_2_] and [P_66614_][124Triz] in acetonitrile (MeCN). The reference electrode was 0.01 mol L^−1^ Ag^+^/Ag formed by dissolving AgNO_3_ in [C_4_mim][NO_3_] and separated from the bulk solution by a glass frit, as reported previously.[18] As well as the reactive IL, [P_66614_][NTf_2_] was examined as a comparison because CO_2_ is unable to chemically bind to this anion. However, by keeping the [P_66614_]^+^ cation consistent, the physically absorbed CO_2_ should be stabilized to the same extent once reduced to the radical anion ([^.^CO_2_]^−^), allowing reduction processes for the chemically and physically absorbed CO_2_ to be distinguished. The CVs recorded at a scan rate of 100 mV s^−1^ at 22 °C in [P_66614_][NTf_2_] and [P_66614_][124Triz] are shown in Figure 1 a and b, respectively. First, the CVs were taken in RTIL solutions saturated with argon (Figure 1). Consistent with previous reports of CVs using metal electrodes in dialkylimidazolium based RTILs, only small capacitive currents are observed in a wide potential window, with no additional features other than the solvent or RTIL reduction in the cathodic range.

**Figure 1:**
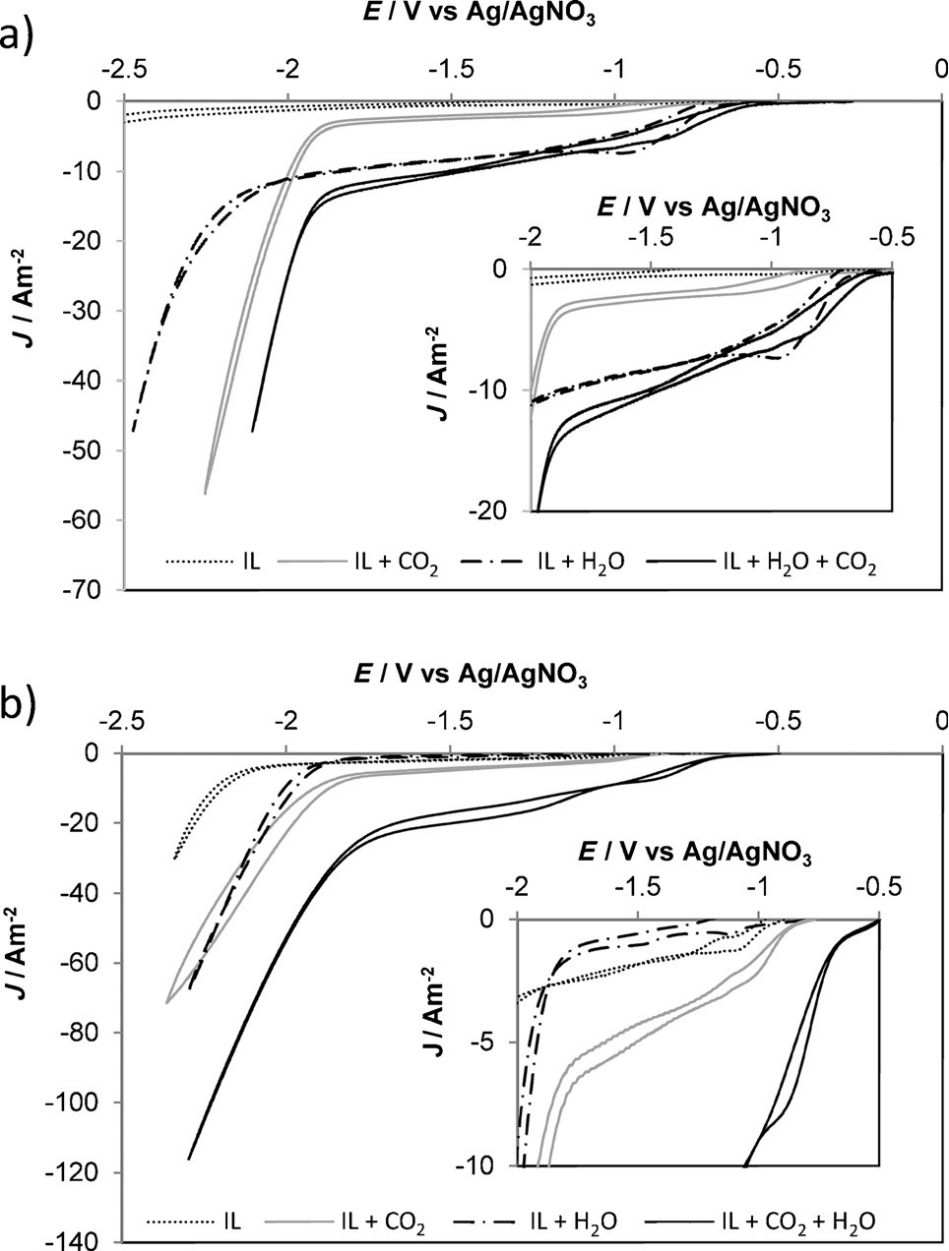
Cyclic voltammograms recorded with a silver working electrode in acetonitrile solutions with 0.1 mol L^−1^ of the ionic liquids (IL) a) [P_66614_][NTf_2_] and b) [P_66614_][124Triz]. Inset: magnified sections.

The CVs taken for solutions of both ionic liquids purged with CO_2_ for 30 min (Figure 1) exhibited cathodic features that can be associated with CO_2_ reduction. As the onset potential is often difficult to define, in agreement with previous reports, the potentials that result in a current density of 6 A m^−2^ were selected as the onset potential for CO_2_ reduction.[11, 19] For the [NTf_2_]^−^-based RTIL, upon the addition of CO_2_, the current starts to increase at −0.9 V followed by a rapid increase at −1.9 V. The rapid increase in current at −1.9 V can be attributed to reduction of physically bound CO_2_, forming [^.^CO_2_]^−^ stabilized by the [P_66614_]^+^ cation. The small current increases from −0.9 V are attributed to trace water contamination (see below). For the [124Triz]^−^-based RTIL, upon the addition of CO_2_, a small current increase is observed at circa −0.9 V and a further small increase is observed at circa −1.5 V; these features are likely to be due to the reduction of CO_2_ bound to the [124Triz]^−^ anion (see below). This is followed by a rapid increase in current at −1.9 V owing to the reduction of physically bound CO_2_ to [^.^CO_2_]^−^ stabilized by the [P_66614_]^+^ cation. This potential is identical to the [NTf_2_]^−^-based RTIL system and is consistent with the [P_66614_]^+^ cation providing the same stabilization to [^.^CO_2_]^−^ in both systems. Owing to the highly hygroscopic nature of RTILs[20] and the high solubility of O_2_ within MeCN, the small features at less negative potential (−0.9 V) are attributed to trace amounts of moisture or oxygen within the system. Prior to this analysis, the RTILs were rigorously dried under high vacuum and purged with Ar for 1 h to limit the influence of adventitious water; however, complete removal is very difficult.

These studies were compared with the response following the addition of H_2_O (0.7 mol L^−1^), the presence of which is required to form protonated reduction products and CO. The presence of H_2_O+CO_2_ within the basic IL should enable the formation of carbonate, which may itself be reduced. CVs of Ar and CO_2_ saturated RTILs in the presence of water are shown in Figure 1. For the [NTf_2_]^−^-based RTIL, the addition of H_2_O to the Ar saturated sample (Figure 1 a) shows a small current increase at circa −0.9 V. This is in the same position that current increases were observed in the anhydrous CO_2_ saturated sample and may suggest the presence of small amounts of moisture in the anhydrous CO_2_-saturated sample. A rapid increase in current is then observed at circa −2.3 V, suggesting the reduction of H_2_O to H_2_ upon the Ag electrode. The addition of H_2_O (0.7 mol L^−1^) to the CO_2_ saturated solution shows a small reduction feature at −0.7 V and a strong cathodic current from −1.9 V. Although a small anodic shift is observed, the responses are similar. The small reduction feature at −0.7 V can be associated with those observed at −0.9 V in the anhydrous CO_2_ saturated sample and in the Ar saturated sample containing H_2_O. The large reduction feature at −1.9 V is identical to that seen in the solely CO_2_ saturated sample and is attributed to the reduction of CO_2_ to [^.^CO_2_]^−^ (Scheme 2, Equation (4)).

**Scheme 2:**
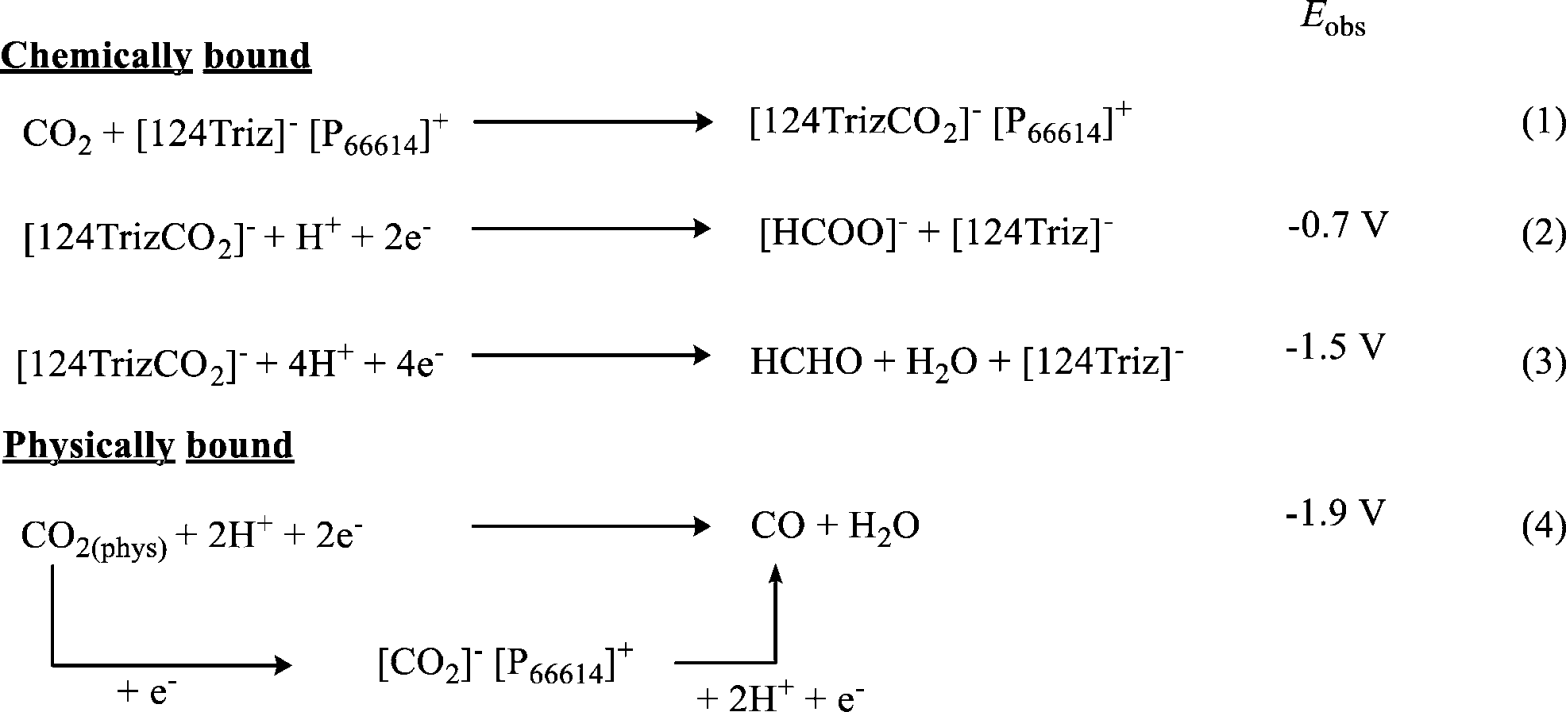
Proposed reduction processes taking place at polarized Ag electrode in ionic liquids saturated with CO_2_.

The addition of H_2_O to the argon-saturated [124Triz]^−^-based RTIL shows two small reduction peaks at circa −1.1 V and −1.5 V followed by a rapid current increase at −1.9 V. The superbasic RTIL, [P_66614_][124Triz] is able to react with H_2_O to form [P_66614_][OH]+[124Triz]−H, the latter of which provides an additional route to H^+^. The first reduction peak at −1.1 V is at a more negative potential than that observed in the anhydrous CO_2_ saturated sample (−0.9 V); however, the second current reduction is in an identical position (−1.5 V). The rapid reduction of current is due to reduction of H_2_O to H_2_. This is at a less negative potential than that observed for the [NTf_2_]^−^ based RTIL (−2.3 V) suggesting the choice of anion has an effect on the H_2_O reduction potential. The most significant changes are observed in the addition of H_2_O (0.7 mol L^−1^) to the CO_2_ saturated [124Triz]^−^ based RTIL, which shows an anodic shift and large enhancement of the two low potential reduction features at −0.7 V and −1.3 V (Figure 1 b) followed by a large increase in current at −1.9 V. The first reduction feature is attributed to the reduction of CO_2_ bound to the [124Triz]^−^ anion to form formate (Scheme 2, Equation (2)). The second reduction feature is also associated with formate product formation via a different mechanism (see below). The third reduction feature, at −1.9 V in Figure 1 b, is assigned to both the reduction of the physically bound CO_2_ stabilized by the [P_66614_]^+^ cation, consistent with the CV observed in [P_66614_][NTf_2_] (Figure 1 a) and the reduction of H_2_O to H_2_. To examine the origin of these features in more detail, an electrolysis study has been performed.

In Scheme 2, Equation (2) indicates that the anion is regenerated and, therefore, should be catalytic. However, any formic acid produced may protonate this anion and the system would not be recyclable. To examine whether the feature at low potential is still present in the presence of the products, one mole equivalent of formic acid to [P_66614_][124Triz] was added to the system (AcN+IL+CO_2_+H_2_O) and the voltammetry compared with the system without formic acid addition (Supporting Information, Figure S1). No reduction in the low potential feature was observed, suggesting no inhibition of the anion binding site. Furthermore, ^1^H NMR spectrum of this sample revealed no evidence for the neutral 124-triazole. It should also be noted that the addition of formic acid to the same system in the absence of CO_2_ does not result in the appearance of peaks below −1 V (not shown).

Electrolysis was performed using a Ag working electrode on the hydrated CO_2_-saturated RTILs within a gas-tight electrochemical cell. For each potential, 10 C of charge was passed before analysis of the products b< ^1^H NMR spectroscopy and online gas chromatography. The variation in product selectivity with applied potential using the RTIL [P_66614_][124Triz] is shown in Figure 2 and the Supporting Information, Table S1. It should be noted that no degradation of the IL was observed in the ^1^H NMR throughout the electrolysis studies.

**Figure 2:**
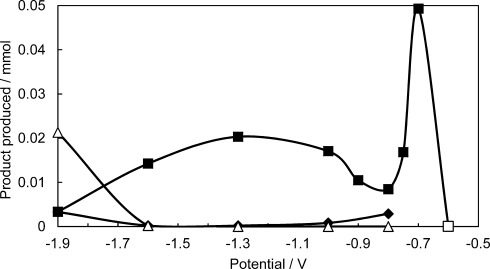
Variation of product yield as a function of applied potential vs Ag/AgNO_3_ after 10 C of charge is passed using a CO_2_ saturated hydrated (0.7 mol L^−1^) 0.1 mol L^−1^ [P_66614_][124Triz] in MeCN electrolyte at a Ag electrode. (▪ formate, ♦ CO, ▵ H_2_). Note the formate generated at −0.6 V (□) was obtained following 24 h of electrolysis during which only 1.5 C of charge had passed.

In [P_66614_][124Triz], formate was the only detectable product in the solution phase. At −0.7 V, 0.05 mmol of formate is produced at a Faradaic efficiency of 95 %. The open circuit potential of the system is −0.53 V; therefore, holding at −0.7 V corresponds to an applied overpotential of 0.17 V. Production of formate at −0.7 V relates to the first reduction peak seen in the CV of the [P_66614_][124Triz]+H_2_O+CO_2_ system. No formate production was observed at less negative applied potentials. A second, albeit smaller, formate maxima is seen at −1.3 V with a Faradaic efficiency of 39 %. This potential corresponds to the second reduction feature seen in the CVs. The formation of formate at −1.3 V is plausibly formed via an alternative mechanism to that seen at −0.7 V, for example by the decomposition of formaldehyde to formate or a radical reaction with superoxide. Further experiments are ongoing to ascertain the origin of the second reduction feature. Finally, at −1.9 V formate production is dramatically reduced and syngas is detected. This corresponds to the rapid increase in reduction current noted on the CV. A summary of the reductive process can be found in Scheme 2.

Formate production is greatly decreased on the onset of CO production, which suggests that reduction of physically absorbed CO_2_ competes with the reduction of chemically bound CO_2_ in this potential range. This may be due to blocking of the electrode surface by adsorbed CO, CO_2_ or other reaction intermediates.

In contrast with the reactive RTIL, electrolysis at −0.7 V in a hydrated CO_2_ saturated [P_66614_][NTf_2_]/MeCN electrolyte showed no formate production. This is consistent with the postulation that no lower energy pathway for CO_2_ reduction is available owing to a lack of CO_2_ binding site on the [NTf_2_]^−^ anion. Furthermore, there is a significant decrease in the production of CO with only 0.2 μmol formed at −1.9 V; that is, over an order of magnitude lower than the amount formed in [P_66614_][124Triz] at 3.3 μmol. This is plausibly due to the higher CO_2_ capacity of [P_66614_][124Triz] over [P_66614_][NTf_2_]. Hydrogen was also detected at −1.9 V with 21.3 μmol detected in [P_66614_][124Triz] and only 6.8 μmol in [P_66614_][NTf_2_] at Faradaic efficiencies of 41.1 and 13.2 %, respectively. The difference can be related to the reduction peak for H_2_ production being more negative for [P_66614_][NTf_2_] (−2.3 V) than [P_66614_][124Triz] (−1.9 V). An alternative explanation for the increased formate production at less negative potentials for the reactive anion system is that the cation, [P_66614_]^+^, is catalytic and, when the anion, [NTf_2_]^−^, is used it acts as a poison to the Ag electrode. To address this issue, [P_66614_][BF_4_] was also examined. Performing electrolysis at −0.7 V resulted in a small amount of formate (0.010 mmol) being produced with a low Faradaic efficiency (19 %). Furthermore, to examine the effect of the cation [N_4444_][124Triz] was also synthesized (see the Supporting Information) and compared with the [P_66614_][124Triz]. Performing electrolysis using this ionic liquid at −0.7 V for 10 C of charge resulted in the production of formate (0.021 mmol) as well as formaldehyde (0.006 mmol) with a combined Faradaic efficiency of 63 %. The contrast of high versus low Faradaic efficiencies and product formation at low potentials when reactive and non-reactive anions are employed, respectively, provides support for the proposal that the reactive anions can provide a new low energy pathway for CO_2_ electroreduction.

In conclusion, the superbasic RTIL [P_66614_][124Triz] has been shown to provide an alternative low-energy pathway for CO_2_ conversion to formate. This is the first time that chemical binding of the neutral CO_2_ molecule to the anion of a RTIL has been shown to decrease the activation energy required for electrochemical reduction. This subsequently leads to the lowest reported applied potentials for CO_2_ reduction to formate on Ag electrodes to date with high Faradaic efficiencies.

## Supporting Information

As a service to our authors and readers, this journal provides supporting information supplied by the authors. Such materials are peer reviewed and may be re-organized for online delivery, but are not copy-edited or typeset. Technical support issues arising from supporting information (other than missing files) should be addressed to the authors.

miscellaneous_information
