# Supplementary material for: Reduction of Carbon Dioxide to Formate at Low Overpotential Using a Superbase Ionic Liquid
Source: Angew Chem Int Ed Engl. 2015 Sep 22;54(47):14164–8. doi: 10.1002/anie.201507629 (PMC4648032; doi:10.1002/anie.201507629)
Supplement: Supplementary file 1 — miscellaneous_information [file anie0054-14164-sd1.pdf]

Angewandte Chemie
Eine Zeitschrift der Gesellschaft Deutscher Chemiker

Supporting Information

Reduction of Carbon Dioxide to Formate at Low Overpotential using a Superbase Ionic Liquid

*Nathan Hollingsworth, S. F. Rebecca Taylor, Miguel T. Galante, Johan Jacquemin, Claudia Longo, Katherine B. Holt, Nora H. de Leeuw, and Christopher Hardacre**

anie_201507629_sm_miscellaneous_information.pdf

Supporting Information

Contents

1.0 Experimental **2**
1.1 Materials and IL synthesis 2
1.2 Electrochemical experiments 2
1.3 Solution phase detection 2
1.4 Gas Phase detection 3
2.0 Results **3**
3.0 Recyclability **3**
4.0 References **4**

1.0 Experimental

1.1 Materials and IL synthesis

Trihexyltetradecylphosphonium chloride ([P_{66614}]Cl, 97.7 %) and Trihexyltetradecylphosphonium tetrafluoroborate (P_{66614}][BF_4] was obtained from Cytec, 1,2,4-triazole (98 %) was purchased from Sigma–Aldrich and lithium bis[(trifluoromethyl)sulfonyl]imide ($LiNTf_2$) purchased from 3M (> 98%). The Ag wire (0.5 mm dia, 99.9985%) and Pt wire (0.3 mm dia, 99.99%) were purchased from Alfa Aesar. Gaseous nitrogen (99.998 %) and carbon dioxide (99.99 %) were obtained from BOC and passed through drying columns before contact with the IL samples. The water was purified using a Milli-Q 18.3 MΩ water system.

[P_{66614}][124Triz] was prepared by a previously reported method[1] using a two step synthesis; [P_{66614}][OH] was synthesized using an anion exchange resin from [P_{66614}]Cl followed by addition of the superbase. [P_{66614}][NTf_2] was synthesised from [P_{66614}]Cl (50.8 g, 0.098 mol) dissolved in dichloromethane 100 ml and added dropwise to $LiNTf_2$ (28.7 g, 0.1 mol)dissolved in distilled water (100 ml) and allowed to stir under ambient conditions overnight. The organic layer was then extracted and washed with distilled water (100 cm^3) repeatedly five times and dried *in vacuo.* [N_{4444}][124Triz] was prepared by equimolar addition of 1,2,4-Triazole in methanol to a 1.0 M tetrabutylammonium hydroxide solution in methanol (Sigma Aldrich). The solution was stirred overnight and dried *in vacuo*. All ionic liquids synthesized were analyzed using ^{1}H-NMR, ^{13}C-NMR and ^{31}P-NMR using a Bruker Avance 400 MHz Ultra shield Plus. and were consistent with previously reported spectra. The cyclic voltammograms for each of these ionic liquids were featureless until the cathodic and anodic breakdown limits. The water content of the ILs was measured using a Metrohm 787 KF Titrino Karl Fischer and was found to be < 0.1 wt% for all ILs synthesised. Halide content was below the detectable limit by $AgNO_3$ testing.

1.2 Electrochemical experiments

Cyclic voltammograms were recorded using an Ecochemie Autolab Potentiostat/Galvanostat (PGSTAT302) and carried out using a three-electrode arrangement with a Ag wire working electrode, a platinum coil as the counter electrode, and all potentials measured with respect to a 0.01 M Ag^+/Ag reference, with $AgNO_3$ dissolved in [C_4mim][NO_3] and separated from the bulk solution *via* a glass frit.

Electrolysis experiments were carried out using a three electrode set up in a sealed 50 cm^3 cell with a gas tight syringe attached. The working electrode was a coiled Ag wire, a Platinum coil was used as the counter electrode (contained within a counter compartment) and potentials were measured with respect to a 0.01 M Ag^+/Ag reference, with $AgNO_3$ dissolved in [C_4mim][NO_3] and separated from the bulk solution via a glass frit. A 0.1 M [P_{66614}][124Triz], 0.7 M H_2O in acetonitrile solution (8 cm^3) was added to the cell and bubbled with CO_2 (25 cm^3/min) for 60 mins, during this time the gas syringe was gradually opened. The cell was then sealed, connected up to a potentiostat (Biologic VMP3 controlled by EC-Lab) and a potential applied to the cell for the time required to pass 10 coulombs of charge.

1.3 Solution phase detection

Solution samples were analysed by taking ^{1}H NMR spectra Bruker Avance III 600 with a DCH cryoprobe. Quantitative analysis was performed using ERETIC2 quantification tool within Topspin 3.2.

1.4 Gas Phase detection

The gas phase of the reaction was analysed using a Perkin Elmer Clarus 500 Gas chromatograph equipped with a TCD and FID (with methanizer) fitted with a packed column (stainless steel, 30 ft, 1/8 inch OD, 2.0 mm ID packed with Haysep DB 100/120 mesh). The gaseous products were transferred to the GC by connecting up the cell and injecting the contents of the gas tight syringe attached to the cell into the GC. H_2 was quantified using the TCD and CO using the FID.

2.0 Results

Table S1. Variation in the Faradaic efficiency in the production of formate, CO and H_2 as a function of applied potential vs Ag/Ag^+ using a CO_2 saturated hydrated (0.7 mol·L^{-1}) 0.1 mol·L^{-1} [P_{66614}][124triz] in MeCN electrolyte at a Ag electrode.

**Electrolysis conditions**	**Product yield and % of Faradaic efficiency**		
	Formate	CO	H_2
-0.6 V	0	-	-
-0.7 V	0.0493 mmol, 95 %	-	-
-0.75 V	0.0168 mmol, 32%	-	-
-0.8 V	0.0085 mmol, 16 %	0.0029 mmol, 6 %	0 mmol, 16 %
-0.9 V	0.0105 mmol, 20 %	-	-
-1.0 V	0.0170 mmol, 33 %	0.0008 mmol, 2 %	0 mmol, 0 %
-1.3 V	0.0203 mmol, 39 %	0.0002 mmol, <0.5 %	0 mmol, 0 %
-1.6 V	0.0142 mmol, 28 %	0.0002 mmol, <0.5 %	0.0002 mmol, 0.4 %
-1.9 V	0.0033 mmol, 6 %	0.0033 mmol, 6 %	0.0213 mmol, 41 %

3.0 Recyclability

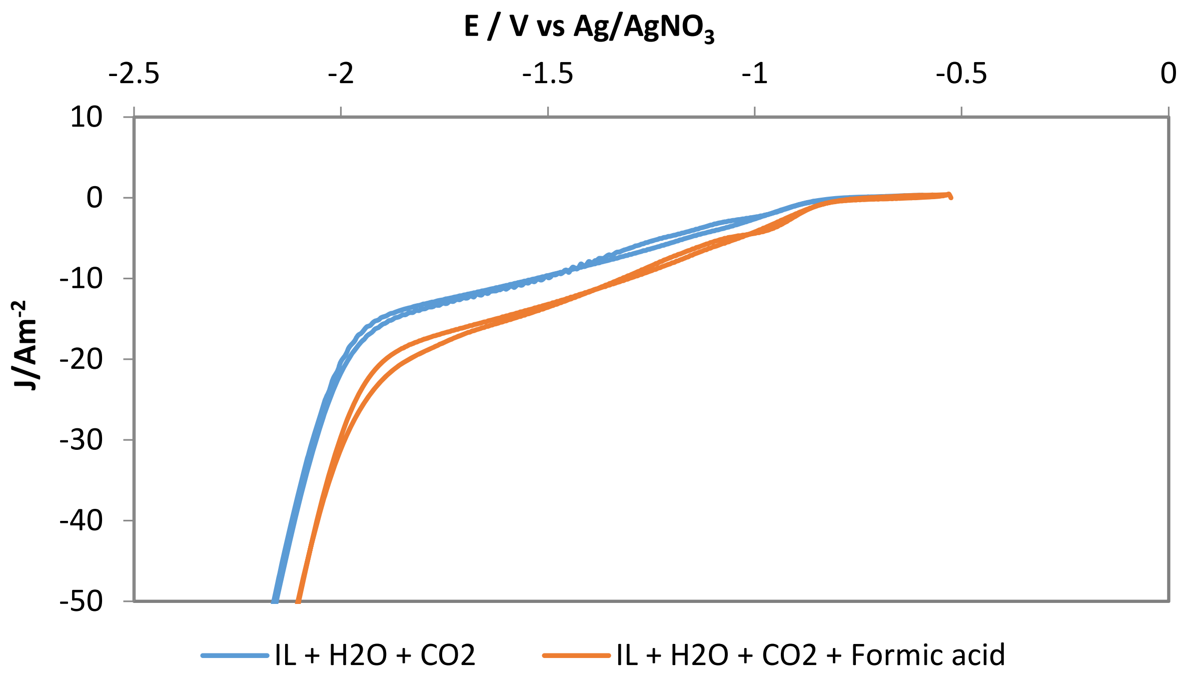

Figure S1 CV of CO_2 saturated hydrated (0.7 mol·L^{-1}) 0.1 mol·L^{-1} [P_{66614}][124triz] in MeCN electrolyte at a Ag electrode with and without the addition of 0.1 mol·L^{-1} formic acid.

4.0 References

[1] S. F. R. Taylor, C. McCrellis, C. McStay, J. Jacquemin, C. Hardacre, M. Mercy, R. Bell, N. de Leeuw, *J Solution Chem* **2015**, 44, 511-527.
